# Visuotactile integration in individuals with fibromyalgia

**DOI:** 10.3389/fnhum.2024.1390609

**Published:** 2024-05-17

**Authors:** Tania Augière, Martin Simoneau, Catherine Mercier

**Affiliations:** ^1^Center for Interdisciplinary Research in Rehabilitation and Social Integration (Cirris), CIUSSS de la Capitale-Nationale, Quebec, QC, Canada; ^2^School of Rehabilitation Sciences, Faculty of Medicine, Laval University, Quebec, QC, Canada; ^3^Department of Kinesiology, Faculty of Medicine, Laval University, Quebec, QC, Canada

**Keywords:** chronic pain, multisensory integration, temporal order judgment, Bayesian approach, body representation, psychophysics

## Abstract

Our brain constantly integrates afferent information, such as visual and tactile information, to perceive the world around us. According to the maximum-likelihood estimation (MLE) model, imprecise information will be weighted less than precise, making the multisensory percept as precise as possible. Individuals with fibromyalgia (FM), a chronic pain syndrome, show alterations in the integration of tactile information. This could lead to a decrease in their weight in a multisensory percept or a general disruption of multisensory integration, making it less beneficial. To assess multisensory integration, 15 participants with FM and 18 pain-free controls performed a temporal-order judgment task in which they received pairs of sequential visual, tactile (unisensory conditions), or visuotactile (multisensory condition) stimulations on the index and the thumb of the non-dominant hand and had to determine which finger was stimulated first. The task enabled us to measure the precision and accuracy of the percept in each condition. Results indicate an increase in precision in the visuotactile condition compared to the unimodal conditions in controls only, although we found no intergroup differences. The observed visuotactile precision was correlated to the precision predicted by the MLE model in both groups, suggesting an optimal integration. Finally, the weights of the sensory information were not different between the groups; however, in the group with FM, higher pain intensity was associated with smaller tactile weight. This study shows no alterations of the visuotactile integration in individuals with FM, though pain may influence tactile weight in these participants.

## 1 Introduction

Our brain constantly integrates the afferent information our senses provide, such as vision, touch, or audition. Numerous studies show that participants generate a more precise perception of an object when they perceive it with several modalities (Gepshtein and Banks, 2003; Alais and Burr, 2004; Bresciani et al., 2006; Nardini et al., 2008; Helbig et al., 2012). The maximum likelihood estimation (MLE) model theorizes how the brain can use noisy afferent information and maintain a stable and coherent perception through multisensory integration (Ernst and Bülthoff, 2004). This model postulates that sensory afferents are weighted according to their reliability: a noisy afferent signal is variable and thus considered less reliable and used less than a reliable signal (Ernst and Banks, 2002; Ernst and Bülthoff, 2004). Ernst and Banks (2002) conducted one of the first studies showing that healthy volunteers integrate sensory information according to the MLE model. In their study, participants had to estimate the height of an object while either seeing it, touching it, or seeing it while touching it (multisensory condition). Participants’ estimations were more precise when visual and tactile information were available than when only visual or tactile information was present, as predicted by the model. This result confirms the benefit of multisensory integration. Moreover, the authors calculated the weight of each sensory information in the visuotactile percept based on the precision of the information (see Method for more details). They showed that, as predicted by the MLE, the more the visual information was degraded (i.e., increasing its variability), the less it was used (i.e., smaller weight) compared to tactile information (Ernst and Banks, 2002). Thereafter, similar results have been reported in several tasks with various modalities (Gepshtein and Banks, 2003; Bresciani et al., 2006; Nardini et al., 2008; Ronsse et al., 2009; Fetsch et al., 2010; Helbig et al., 2012; Chancel et al., 2016; Sexton et al., 2019). This way of integrating information seems essential to generate a coherent body representation, as shown by studies in which the degradation of somatosensory information of a given body part is accompanied by perturbations in the representation of this body part (Lackner, 1988; de Vignemont et al., 2005; Chancel and Ehrsson, 2023).

One way to study sensory information integration is with temporal-order judgment (TOJ) tasks (Morein-Zamir et al., 2003; Ley et al., 2009; Maiworm and Röder, 2011). In these tasks, participants must judge the order of two successive stimuli. The delay between the stimuli varies, and psychometric functions can be generated to describe the participants’ answers according to the delay. Two outcome variables are then extrapolated from the function: the variability (SD, standard deviation) and the accuracy (M, mean) of the percept. Stimuli can be unisensory (e.g., two sequential visual or tactile stimuli) or multisensory (e.g., two sequential visuotactile stimuli). The precision and the accuracy of the multisensory percept are compared to the precision and the accuracy of the unisensory percepts, and the weight of each sensory modality in the multisensory percept can be calculated. In a study using a TOJ task with pairs of auditory, tactile, or audiotactile stimuli, tactile stimuli were degraded to test the effect of different noise levels on audiotactile integration (Ley et al., 2009). Using psychometric functions, the authors found that the empirical data followed the MLE model, with a more precise percept in the multisensory condition compared to the unisensory percepts and with an effect of the increased tactile noise on the weight attributed to tactile information (Ley et al., 2009). This suggests an optimal (i.e., according to the MLE model) integration of artificially noisy sensory information in healthy participants. But what if sensory information is noisy for a long rather than a short period? Does multisensory integration remain optimal in such conditions? Studying multisensory integration in clinical populations that show unisensory integration alterations can help answer these questions.

People with chronic pain often show alterations of the unisensory integration of somatosensory stimuli, such as a hypersensitivity (i.e., lower detection threshold; Westermann et al., 2011; Achenbach et al., 2020) or hyposensitivity (i.e., higher detection threshold; Rommel et al., 2001; Geber et al., 2008; Maier et al., 2010; Hochman et al., 2013; Hilgenberg-Sydney et al., 2016; Evdokimov et al., 2019; Sobeeh et al., 2023) to tactile stimuli, and a worse tactile acuity (Pleger et al., 2006; Harvie et al., 2017; Martínez et al., 2019; Kim et al., 2020; López-de-Uralde-Villanueva et al., 2020). These alterations are sometimes accompanied by distortions of the body representation (Moseley, 2008). In a study in which tactile perception was measured with the two-point discrimination (TPD) test, individuals with low back pain had a worse perception (i.e., higher threshold) than pain-free controls at their lower back (Moseley, 2008). Moreover, when they were asked to draw their lower back as they felt it (as opposed to how they saw it), the drawings were distorted compared to the control group (Moseley, 2008). Alterations of the body representation in chronic pain populations can manifest themselves as a perception of changes in the size (Lewis et al., 2007; Lotze and Moseley, 2007; Peltz et al., 2011; Valenzuela-Moguillansky, 2013; Dieguez and Lopez, 2017; Brun et al., 2019; Menten et al., 2022; Scandola et al., 2022), the shape (Moseley, 2008; Valenzuela-Moguillansky, 2013; Menten et al., 2022), the weight (Valenzuela-Moguillansky, 2013; Brun et al., 2019), the existence (Moseley, 2008; Valenzuela-Moguillansky, 2013; Dieguez and Lopez, 2017; Menten et al., 2022) of the body part or in the feeling of ownership over the body part (Lewis et al., 2007; Valenzuela-Moguillansky, 2013; Scandola et al., 2022). These body representation distortions could stem from alterations in the multisensory integration of somatosensory information in individuals with chronic pain. Indeed, according to the MLE model, somatosensory information should be considered unreliable in these populations and, therefore, used less (i.e., smaller weight) than other sensory information, such as vision. This weighting strategy could allow for the generation of precise perceptions based on noisy somatosensory signals.

Fibromyalgia (FM) represents an excellent model to test this hypothesis. This syndrome affects about 2–3% of the population (Branco et al., 2010; Wolfe et al., 2013) and is defined by widespread chronic pain accompanied by various symptoms affecting sleep, cognition, and motricity (Häuser et al., 2015; Häuser and Fitzcharles, 2018). People with FM also show signs of alterations of the unisensory integration of somatosensory information, such as higher tactile detection thresholds (Üçeyler et al., 2015; Evdokimov et al., 2019), higher TPD thresholds (i.e., poorer tactile acuity; Martínez et al., 2019), and referred sensations (i.e., sensations arising from outside the stimulated site; Martínez et al., 2019) following tactile stimulation. In two recent studies, participants with FM and pain-free participants received pairs of temporally close tactile stimuli on the hand and were asked to indicate when they perceived them as two separate stimuli (Gunendi et al., 2019; Tertemiz and Tepe, 2022). In both studies, participants with FM needed inter-stimuli delay on average two to four times longer than the controls to perceive the stimuli as separate. Moreover, a correlation was found between the altered temporal discrimination threshold and several FM symptoms, such as the intensity of pain, the severity of various somatic and cognitive symptoms, and the level of impairment (Gunendi et al., 2019). In addition to these alterations, individuals with FM experience body representation distortions (Valenzuela-Moguillansky, 2013; Menten et al., 2022; Scandola et al., 2022), which could stem from modifications of the weighting of somatosensory information. However, no study has directly tested multisensory integration in this population.

This study aimed to assess visuotactile integration in people with FM compared to pain-free individuals (CTRL). **Objective 1** was to evaluate the precision and accuracy of the unisensory percepts (visual, tactile) and compare them across groups. We expected that the participants with FM would show less precision and accuracy in the tactile percept than the visual percept compared to the CTRL participants. **Objective 2** was to assess the benefit in precision and accuracy of having access to several sources of sensory information and compare this benefit across groups. We hypothesized that CTRL participants would exhibit a stronger benefit in precision, and possibly in accuracy, than FM participants. **Objective 3** was to determine the relationship between the *observed* precision of the multisensory percept and the precision *predicted* by the MLE model in individuals with FM and CTRL participants. We expected a positive correlation between the observed and the predicted precision in CTRL participants but not in participants with FM, suggesting a sub-optimal visuotactile integration in the latter group. Finally, **Objective 4** was to compare the weight given to each source of sensory information in the FM group vs. the CTRL group. We expected that participants with FM would rely less on tactile information, as shown by a smaller weight attributed to this information, compared to the CTRL group.

## 2 Materials and methods

### 2.1 Participants and ethical statement

We recruited twenty participants with FM via Laval University, the Fibromyalgia Association of Quebec City, the Quebec Research Pain Network, and the Fibromyalgia Association of Montreal. Twenty-four healthy controls, matched to the FM group for age and sex, were recruited via Laval University, the FADOQ Network (a group of organizations for residents of Quebec who are ≥55 years old), and Facebook. The sample size was based on pragmatic considerations of how many participants with FM would be willing to participate given the fatigue, pain, and brain fog associated with this syndrome. The inclusion criteria for all the participants were: (1) being ≥18 years old; (2) having a normal or corrected-to-normal vision; (3) having no non-neurological sensitive alterations (burns, bruises, etc.) on the index and thumb of the non-dominant hand; (4) having no neurological disorders.

We included participants with FM if: (1) they had received a diagnosis of FM according to the American College of Rheumatology by a qualified doctor (Wolfe et al., 1990, 2010, 2016); (2) they had not undergone surgery in the last three months. CTRL participants were excluded if they had a history of chronic pain or acute pain severe enough to interfere with daily functioning in the previous month or of acute pain on the day of the participation. All participants provided their written informed consent before they participated in this study. The experiment was performed following the Declaration of Helsinki, and the study protocol was approved by the local ethical review board (CIUSSS de la Capitale-Nationale, Quebec City, Canada, no 2022-2334 RIS).

### 2.2 Study design

Participants took part in two experimental sessions at the Center for Interdisciplinary Research in Rehabilitation and Social Integration. In the first session, all participants filled out a manual laterality questionnaire, and participants with FM were questioned about their medical history and asked to fill out a clinical questionnaire. Another study, not reported here, was conducted during that first session (article in preparation). In the second session, lasting about two hours, the participants performed the visuotactile TOJ task, in which they received two successive stimulations on the index and thumb of their non-dominant hand and had to determine which one they perceived first. The stimulations were either visual, tactile, or visuotactile and were performed in a randomized order. The two sessions were conducted with a delay of between 0 and 9 days.

### 2.3 Questionnaires

All participants completed the French version of the Edinburgh Handedness Inventory (Oldfield, 1971; Ransil and Schachter, 1994; Veale, 2014) to determine their manual preference. Participants with FM were questioned about their medical history and asked to fill out the French version of the Brief Pain Inventory (BPI; Cleeland, 1991) to assess the severity of their pain and its impact on daily function.

### 2.4 TOJ visuotactile task

Participants sat on a comfortable chair, with their non-dominant arm pronated on a table. The two stimulation sites, site 1 and site 2, were located on the dorsal part of the thumb and index finger, respectively. Two pieces of paper placed next to the fingers indicated the name of each site (1 or 2). The fingers were separated by 55 mm. Two stickers glued on the table indicated the position of the fingers so the participants could move the hand during the breaks and replace their fingers in the same position easily. The stimulations were either tactile (T), visual (V), or both visual and tactile (visuotactile condition; VT). The first two conditions (T and V) were unisensory, meaning only one sensory modality was integrated, whereas the VT condition was multisensory. The tactile stimulations were two non-painful electrical stimulations provided by a Digitimer DS7A via four self-adhesive bipolar electrodes (two for the thumb and two for the index finger) (EL504, Biopac Systems Inc.). Electrical stimulations on the skin surface are often used as an easily controllable and non-invasive way to administer tactile stimulation, as it activates tactile receptors (Chouvardas et al., 2008; Marcus and Fuglevand, 2009; Yem and Kajimoto, 2017). Moreover, tactile perception seems similar between mechanical and electrical stimulations (Marcus and Fuglevand, 2009). The intensity of the stimulations was set for each participant by averaging the detection threshold of the two fingers and multiplying it by 1.5. We determined the detection threshold by continuously increasing the intensity of the electrical stimulation by steps of 0.1 mA, starting at 0.1 mA. We instructed the participants to say when they felt something. The goal was to determine an easily detectable intensity that was not painful or uncomfortable for both fingers. If this was not the case, the intensity was increased or decreased by steps of 0.1 mA until the participant confirmed it was. The intensity was increased by an average of 0.2 mA for six participants and decreased by 0.03 mA for one participant. The duration of the stimulations was 1 ms, and the voltage was 200 V. The visual stimulations consisted of two dim red LED attached to the dorsal part of the fingers. The flashes of light lasted 10 ms. The visuotactile stimulations were defined as the co-occurrence, on each stimulation site, of both tactile and visual stimulations.

We randomized the order of the stimulation (i.e., which site is stimulated first) and the order of the conditions (V, T, VT). In each condition (V, T, VT), two stimulations, one on each stimulation site, were performed sequentially with a variable stimulus onset asynchrony (SOA) between the two. The SOA corresponded to the time between the emission of the first stimulation and the emission of the second stimulation and could vary across seven values (0, ±10, ±25, ±50, ±100, ±300, ±800 ms). By convention, a positive SOA meant that site 1 was stimulated first, and a negative SOA meant that site 2 was stimulated first. For a SOA equal to 0 ms, the sites were stimulated simultaneously.

A familiarisation phase of at least six trials (3 per condition) with a SOA of 1,250 ms was first performed until the participants felt comfortable with the task. The test phase was composed of 130 trials per condition, for a total of 390 trials. We offered a break every 20 to 23 trials and asked the participants if the stimulations were still detectable and not painful or uncomfortable. We also asked the participants with FM to rate the pain intensity of their non-dominant upper limb on a 0–10 visual analog scale (0 = no pain; 10 = worst imaginable pain). Moreover, participants could ask for additional breaks throughout the task.

### 2.5 Data preprocessing

According to the SOA, answers were expressed as the frequency of answers: “site 1 was stimulated first.” For each participant and condition, we preprocessed participants’ answers using Matlab (version R2022b, MathWorks Inc., Natick, MA, United States) with a script Clark and colleagues wrote (Clark and Merfeld, 2021). The script identified outliers and fitted a psychometric function on the data for later interpolating the outcome variables. Outliers were defined as trials whose influence on the main variables was the most significant (chances of occurring was *p* < 0.01) if they were to be removed (Clark and Merfeld, 2021). If the search for outliers found more than one trial fitting this description, only the most inconsistent data point was removed. Additional searches with the remaining trials were ran until no trial reached the statistical criterion. This method of outlier identification and removal yielded accurate estimates of the outcome variables across a wide range of SOA (Clark and Merfeld, 2021).

**Figure 1:**
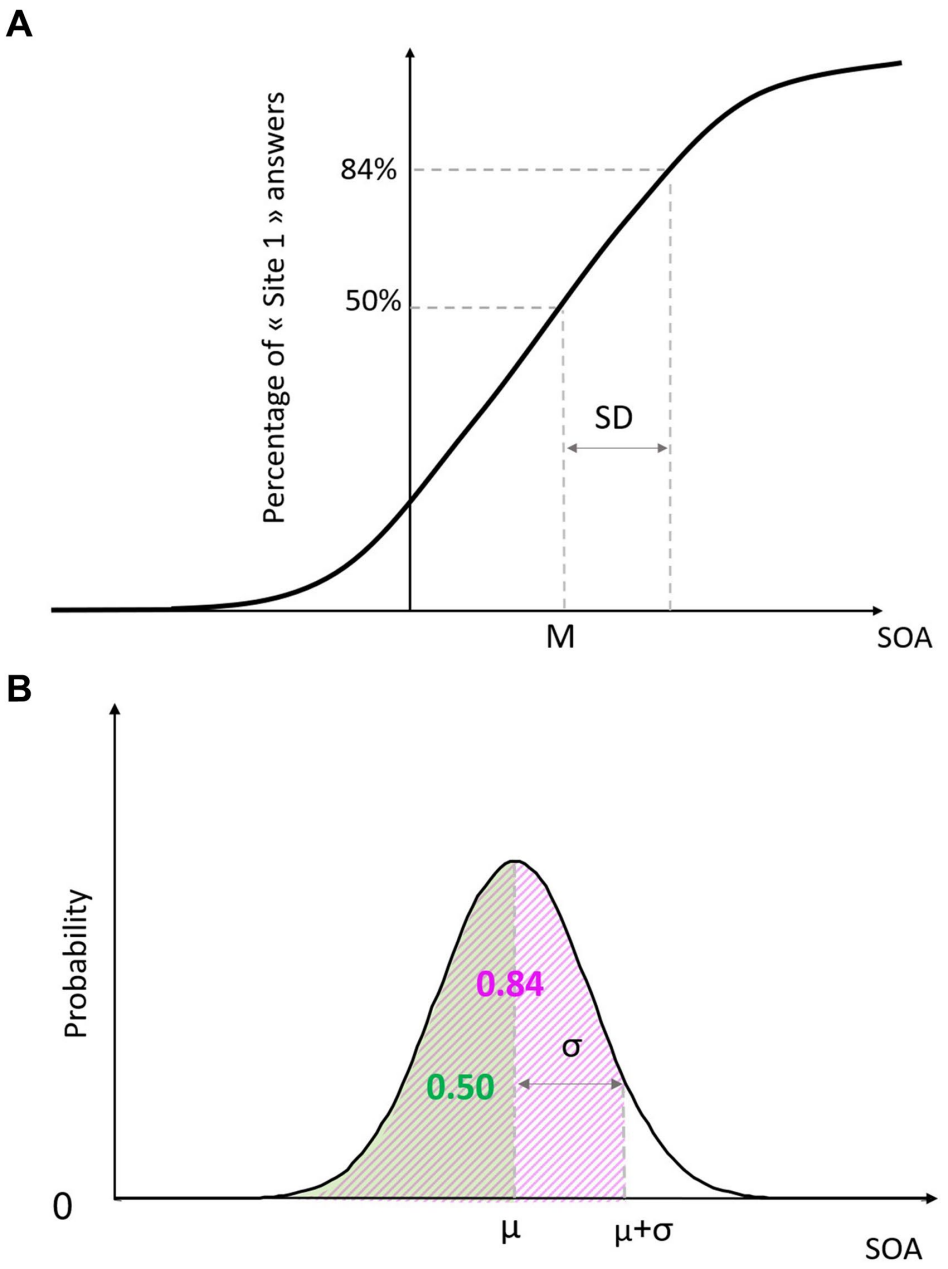
Psychometric function **(A)** and Bayesian function **(B)**. SOA, stimulus onset asynchrony.

The two outcome variables interpolated from the psychometric function were the accuracy M and the variability SD. These variables correspond to the underlying parameters of the Bayesian function. Examples of psychometric and Bayesian functions are shown in Figure 1. We defined M as the SOA for which the participants perceived the two stimuli as simultaneous (i.e., X_0.50_). It corresponds to the mean μ of the Bayesian function. The variability SD is the difference between the mean X_0.50_ and X_0.84_, the point distant from one standard deviation to the mean of the Bayesian function (i.e., σ). The higher the SD is, the flatter the curve is, and the poorer the discrimination abilities are. For each Condition and Group, the means of M and SD were interpolated from the psychometric curve and used for Bayesian modeling.

For each group, according to the SOA, we averaged the percentage of answers for “Site 1” and fitted the psychometric curves to evaluate the performance during the unisensory condition.

For the multisensory condition (VT), we calculated the benefit in precision (BP) and accuracy (BA) and compared the results to the best unisensory condition (V or T), as follows:

(1) $$BP=\frac{\min\left(SD_{V\;\mathrm{or}\;T}\right)-SD_{VT}}{\min\left(SD_{V\;\mathrm{or}\;T}\right)}$$

(2) $$\mathrm{BA}=\frac{\min\left(∥M_{V\;\mathrm{or}\;T}∥\right)-∥M_{VT}∥}{\min\left(∥M_{V\;\mathrm{or}\;T}∥\right)}$$

A positive value means an improvement (i.e., in precision for the BP and accuracy for the BA) in the VT condition (i.e., a benefit of multisensory integration); a negative value reflects a degradation of the performance in the VT condition (Chancel et al., 2016).

To ensure multisensory integration was possible (i.e., to avoid floor or ceiling effects), the analyses excluded participants who scored lower than 60% or higher than 90% success on any unisensory condition. A score too low means they did not perceive the sensory signal well and answered by chance; therefore, they had no advantage of using it in the VT condition. A score that is too high indicates that they could not improve with the additional sensory information in the VT condition (Rohde et al., 2016; Regenbogen et al., 2018).

### 2.6 Bayesian modeling

The MLE model was used to predict the multisensory percept’s value (μ_VT_) and variability (σ_VT_) if the integration was optimal, given the unisensory percepts’ values (M_T_ and M_V_) and variabilities (SD_T_ and SD_V_).

We calculated the predicted visuotactile percept as follows:

(3) $$\mu_{VT}=w_{V}M_{V}+w_{T}M_{T}$$

with w, the weight attributed to each sensory modality, according to their reliability r:

(4) $$w_{V}=\frac{r_{V}}{r_{V}+r_{T}}$$

(5) $$r=\frac{1}{SD^{2}}$$

We determined the variability of the visuotactile percept by computing the square root of σ^2^_VT_:

(6) $$\sigma_{VT}^{2}=\frac{SD_{V}^{2}SD_{T}^{2}}{SD_{V}^{2}+SD_{T}^{2}}$$

According to the MLE model, if the integration of the visual and the tactile cues is optimal, SD_VT_ should be positively correlated to σ_VT_.

### 2.7 Statistics

#### 2.7.1 Clinical and demographic data

To ensure we adequately age-matched participants, we compared ages between the groups using the Mann–Whitney U test. Tactile detection thresholds were also compared between the groups with a Mann–Whitney U. All other clinical and demographic data were synthesized with descriptive statistics.

#### 2.7.2 Visuotactile integration data

Since none of the data followed a normal distribution (as shown by a significant Shapiro–Wilk test), and transformations did not resolve the skewness of the data, we used non-parametric tests.

For **Objective 1**, a nparLD, a non-parametric equivalent of a repeated measures ANOVA (Noguchi et al., 2012), was performed on M and SD, with the within-subject factor Condition (V, T) and the between-subject factor Group (FM, CTRL). NparLD is a robust method for mixed designs with inequivalent samples and does not require normality of distributions and homoscedasticity (Noguchi et al., 2012). For **Objective 2**, the 95% confidence intervals of the BP and the BA were calculated to assess whether the benefits in precision and accuracy were positive for each group (FM, CTRL). Then, we compared the BP and the BA between the groups with a Mann–Whitney U. For **Objective 3**, we evaluated the optimality of the visuotactile integration by testing the correlation between the precision of the visuotactile percept that was observed (i.e., observed SD_VT_) and the one that was predicted by the MLE (i.e., optimal SD_VT_), using a Spearman correlation. Finally, for **Objective 4**, the weight given to the tactile information was compared between the two groups (FM, CTRL) using a Mann–Whitney U test. Since the weight attributed to the visual information could be inferred from the weight of the tactile information (w_V_ = 1 – w_T_), we performed only one comparison. The statistical tests were performed with IBM SPSS (version 29), except for the nparLD, which was conducted with Rstudio Team (2023).

Note that because we used non-parametric statistics, the data reported include median and interquartile range (IQR). Comparisons were considered statistically significant for a *p* < 0.05.

## 3 Results

Of the 44 participants (20 participants with FM and 24 pain-free controls) recruited, we excluded 11 participants either because (1) their performance in at least one of the unisensory conditions was not in the predetermined range of [60; 90]% (*n* = 6); (2) the SD was abnormally high in a modality even though the performance score was within the predetermined range (*n* = 2); (3) the nonlinear curve did not fit the data (*n* = 1); or (4) the participant discontinued their participation (*n* = 1). Of the participants whose performance was outside the range, seven scores were below 60% (6 CTRL participants: 4 in the Tactile condition and 2 in the Visual condition; and 1 participant with FM in both the Tactile and the Visual condition) and 1 was above 90% (1 participant with FM, in the Visual condition). In total, the data of 33 participants (15 participants with FM and 18 CTRL) was used for further analysis.

### 3.1 Clinical and demographic data

The FM group was composed of 14 females and 1 male and the CTRL group of 17 females and 1 male. The groups did not differ in age (FM: median = 45.0, IQR = 13.0 years old; CTRL: median = 38.0, IQR = 28.0 years old; *p* = 0.53). Tactile detection thresholds were not different between the groups (FM: median = 0.80 mA, IQR = 0.40 mA; CTRL: median = 1.05 mA, IQR = 0.33 mA; *p* = 0.244). The clinical characteristics of the FM group are presented in Table 1.

**Table 1:** Clinical characteristics of participants with FM.

Participant	Sex	Age (years)	Currently working?	Pain duration (years)	BPI: pain severity	BPI: pain interference	Current comorbidities	Pharmacological treatments	Non-pharmacological treatments
S01	F	25	Yes	11	6.0	2.7	Attention deficit disorder, asthma, hypothyroidism, irritable bowel syndrome, migraines	Lyrica	
S02	F	35	Yes	23	4.0	5.0	Arthritis, post-traumatic stress disorder, migraines, occipital neuralgia, morbid obesity	Acetaminophen, lyrica, effexor, cyclobenzaprine, voltaren	
S04	F	47	Yes	33	7.0	3.3	Irritable bowel syndrome	Amitriptyline, pregabalin, acetaminophen, naproxen	Physiotherapy
S05	F	40	Yes	40	1.8	2.7		Robax-platinum, acetaminophen	Chiropractic, massotherapy, osteopathy
S12	F	31	Yes	8	6.3	4.7	Rheumatoid arthritis, Crohn’s disease, endometriosis	Lyrica, acetaminophen	
S14	F	47	No	32	5.3	3.3		Tramadol, duloxetine, naproxen	Massotherapy, physiotherapy
S15	F	56	Yes	30	4.3	2.4	Hypothyroidism	Pregabalin, duloxetin	Chiropractic, physiotherapy, osteopathy, massotherapy
S16	F	35	Yes	2	4.5	3.1			Massotherapy
S22	F	45	Yes	38	5.0	5.7	Generalized anxiety disorder, irritable bowel syndrome	Citalopram, THC oil	Chiropractic
S23	M	66	No	20	7.0	3.3		Duloxetine	Massotherapy
S25	F	43	Yes	10	5.3	4.4	Arthritis, migraines	Ibuprofen, acetaminophen, robaxacet	
S34	F	57	No	25	6.5	5.0	Meniere’s disease (deafness of right ear), overactive bladder, severe allergies	Cymbalta, naproxen, amitryptyline	Osteopathy, chiropractic, zootherapy
S36	F	53	Yes	8	5.8	6.1	Hypothyroidism, irritable bowel syndrome, sinus arrhythmia, gastric reflux	Cymbalta, naproxen	Psychotherapy, massotherapy, reiki
S37	F	42	Yes	33	5.8	8.7	Depression and suicidal thoughts	Voltaren, naproxen, cannabis	
S43	F	48	Yes	2	2.8	2.4	Hypothyroidism, asthma, sleep apnea	Amitriptyline, duloxetine, cyclobenzaprine	Massotherapy, osteopathy, acupuncture, self-hypnosis
Median ± IQR				21.5 ± 25.0	5.3 ± 1.8	4.3 ± 2.0			

F, female; M, male; BPI, brief pain inventory; IQR, interquartile range.

### 3.2 Visuotactile TOJ task

The algorithm excluded fifty-four trials (which accounts for 0.008%, as the total number of trials was: 130 trials × 3 conditions × 33 participants = 694,980 trials) because they were considered outliers.

**Figure 2:**
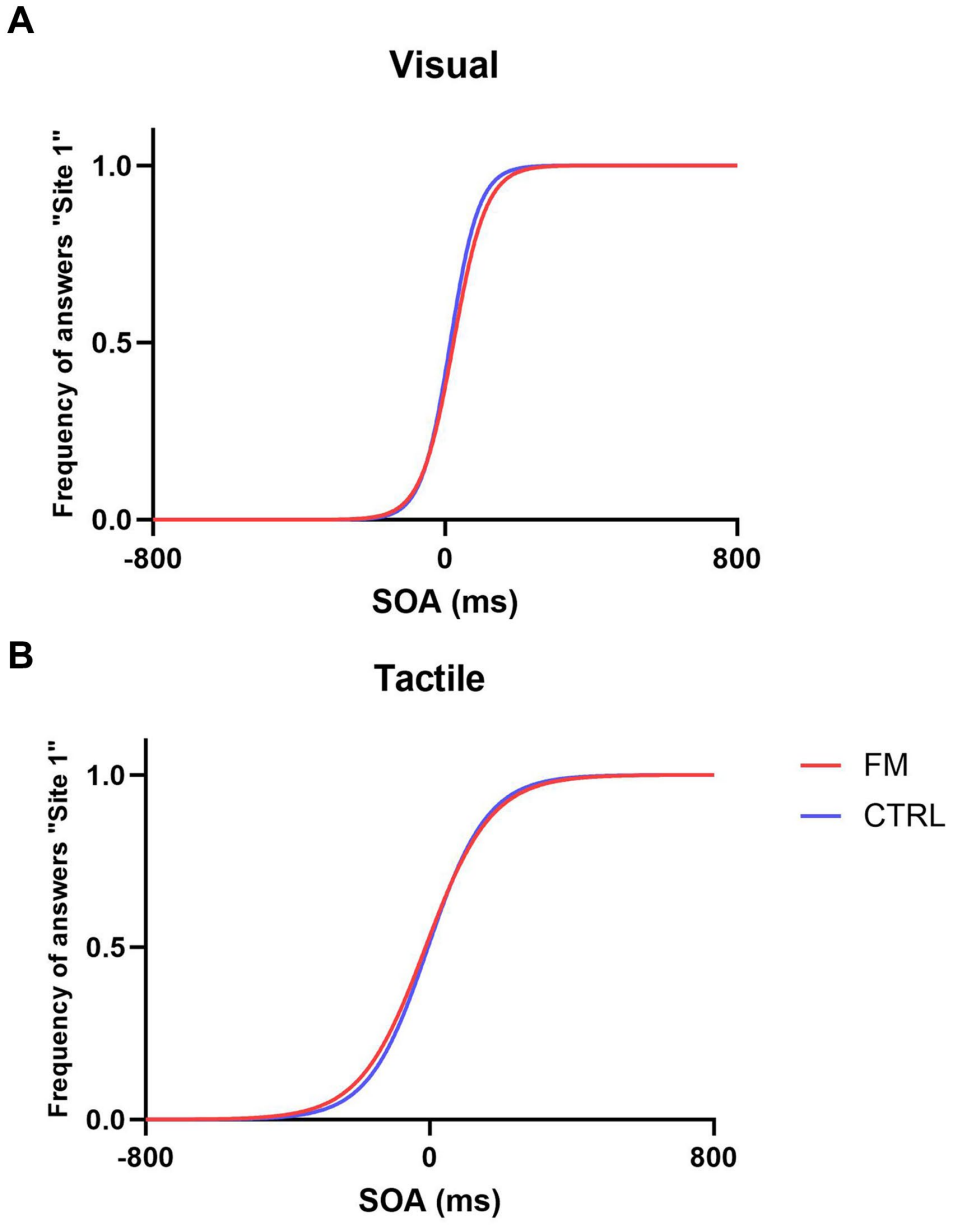
Psychometric functions in the Visual Condition **(A)** and Tactile Condition **(B)** for the FM group (in red) and for the CTRL group (in blue).

The psychometric functions for each group in the two unisensory conditions are presented in Figure 2.

For **Objective 1**, the nparLD showed that the precision of the tactile percept was not statistically different from the precision of the visual percept (*F*(1,32) = 1.04, *p* = 0.31). No effect of the Group (*F*(1,32) = 0.81, *p* = 0.37) and no interaction were found (*F*(1,32) = 0.13, *p* = 0.72). The accuracy of the tactile percept was better than the accuracy of the visual percept for both groups (for FM, in Tactile: median = −8.33, IQR = 74.60; in Visual: median = 19.27, IQR = 80.28; for CTRL in Tactile: median = −12.28, IQR = 69.26; in Visual: median = 12.30, IQR = 22.88; *F*(1,32) = 6.98, *p* < 0.01). No effect of the Group (*F*(1,32) = 0.91, *p* = 0.34) and no interaction were found (*F*(1,32) = 0.18, *p* = 0.67).

**Figure 3:**
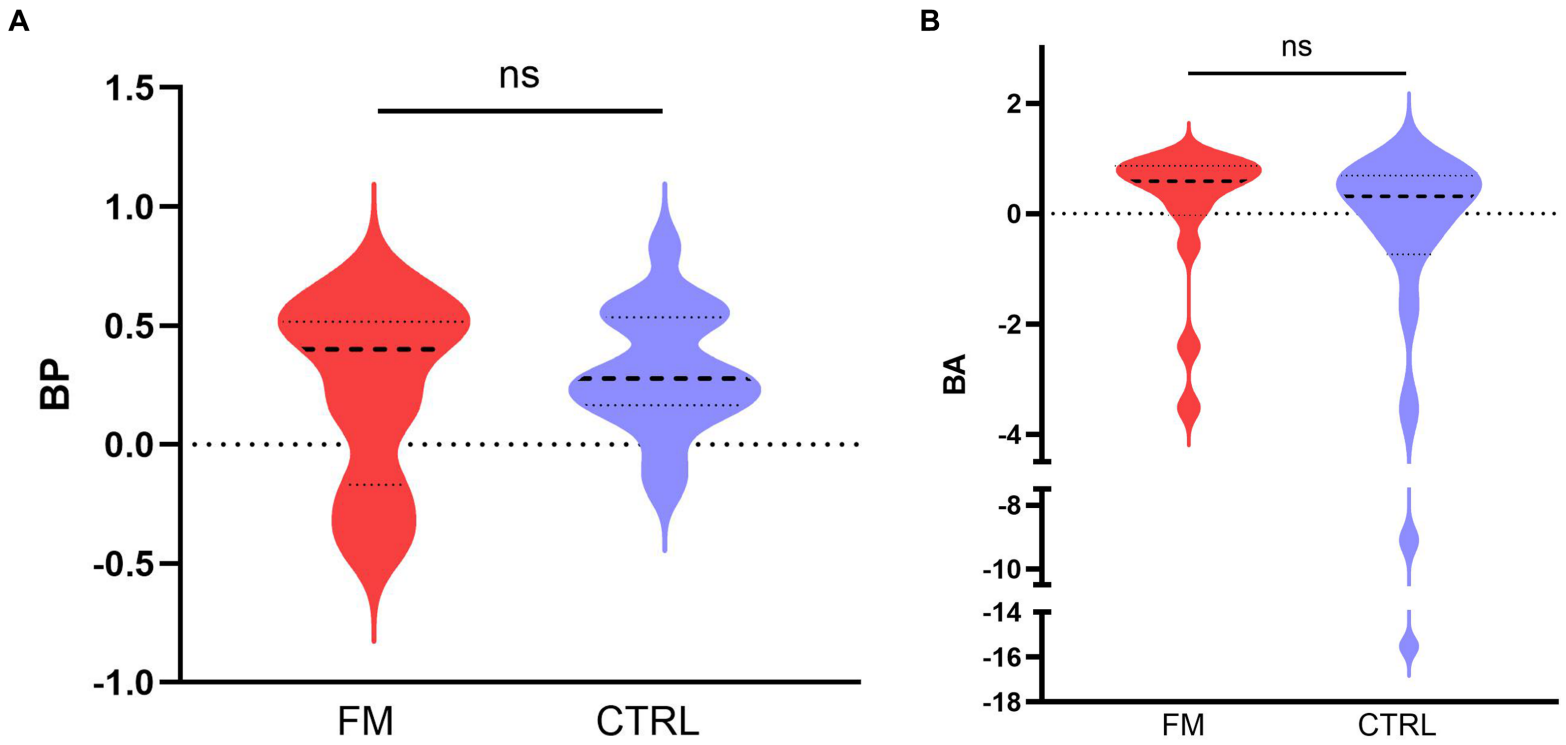
Median (bold discontinued line) benefit in precision **(A)** and in accuracy **(B)**, for the CTRL (in blue) and the FM (in red) group. The thin discontinued lines correspond to the 95% confidence intervals. FM, participants with fibromyalgia; CTRL, pain-free participants; ns, non-significant.

According to **Objective 2**, we compared the BP and the BA between the two groups (FM, CTRL). The results are depicted in Figure 3. The 95% confidence interval indicates a positive BP for the CTRL group but not for the FM group. This result suggests that, in CTRL participants, the visuotactile percept was more precise than the most precise unisensory percept, which is a sign of a visuotactile integration. The Mann–Whitney U test showed no difference in BP between the groups (FM: median = 0.41, IQR = 0.54; CTRL: median = 0.28, IQR = 0.35; *p* = 0.762). The absence of an increase in precision for the FM group, even though no difference in BP was observed between the groups, could be due to higher variability in the FM group, which was composed of fewer participants than the CTRL group. Moreover, a slight bimodal distribution could be observed for the FM group; this will be commented on in the results for Objective 4.

For the BA, we found no difference between the groups (FM: median = 0.59, IQR = 0.73; CTRL: median = 0.31, IQR = 1.27; *p* = 0.119), and the 95% confidence interval included the zero for both groups, although we observed a tendency towards a benefit in accuracy for the FM group (IC95% = [−0.0006; 0,8,494]). This result indicates that the multisensory percept tended to be more accurate than the unisensory percepts for the FM group but not for the CTRL group.

**Figure 4:**
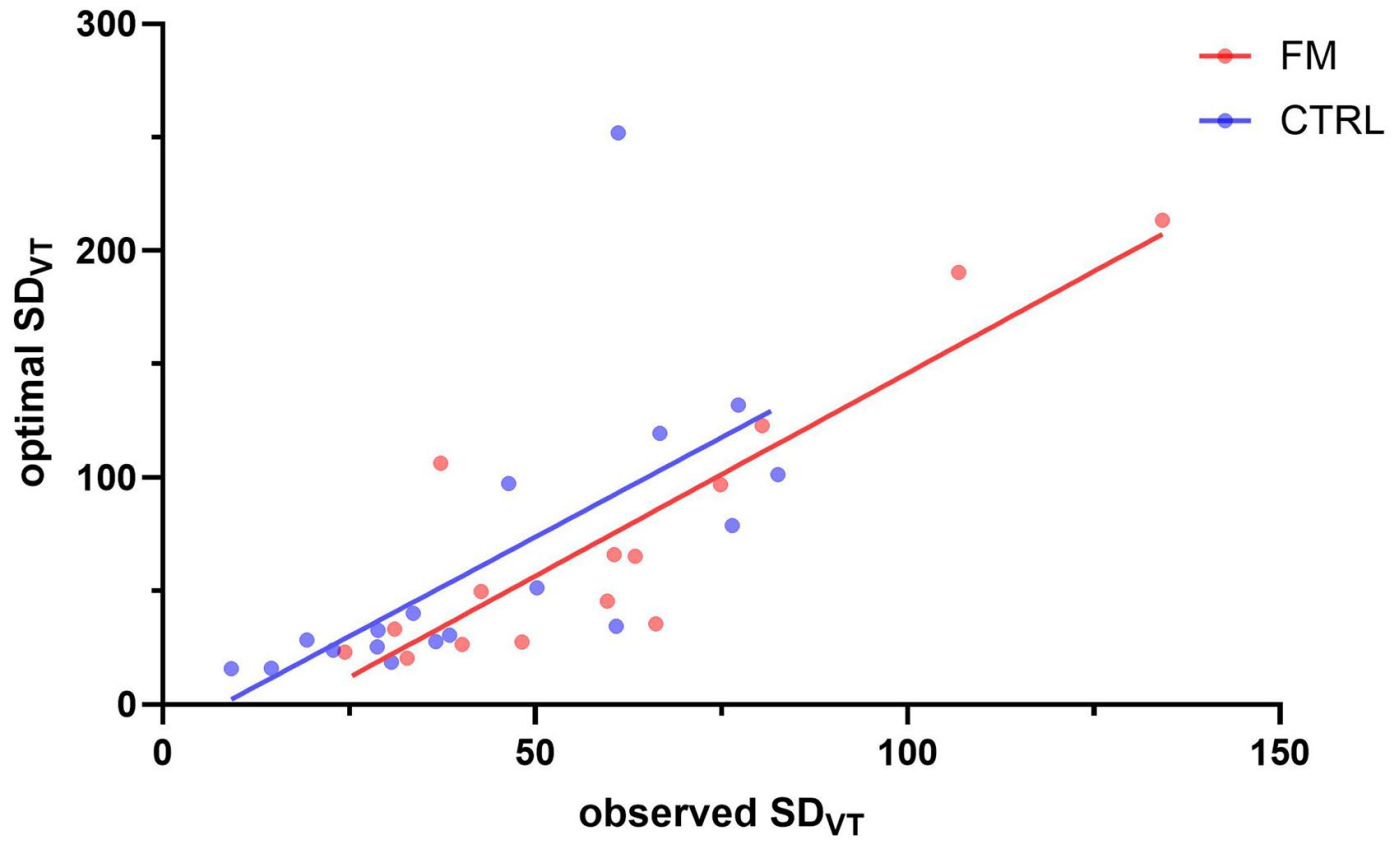
Correlation between the observed SD_VT_ and the SD_VT_ predicted by the MLE. The red and blue dots represent participants of the FM and CTRL group, respectively. The lines illustrate the correlation between the observed SD_VT_ and the optimal SD_VT_, for the FM (in red) and for the CTRL (in blue) participants. FM, participants with fibromyalgia; CTRL, pain-free participants; VT, visuotactile.

The results for **Objective 3** are presented in Figure 4. Spearman’s test revealed that the observed variability of the visuotactile percept (observed SD_VT_) and the variability as predicted by the MLE (optimal SD_VT_) were positively associated for the CTRL (Spearman’s ρ = 0.880; *p* < 0.001) and the FM group (Spearman’s ρ = 0.779; *p* < 0.001). These results indicate an optimal integration of visual and tactile information.

**Figure 5:**
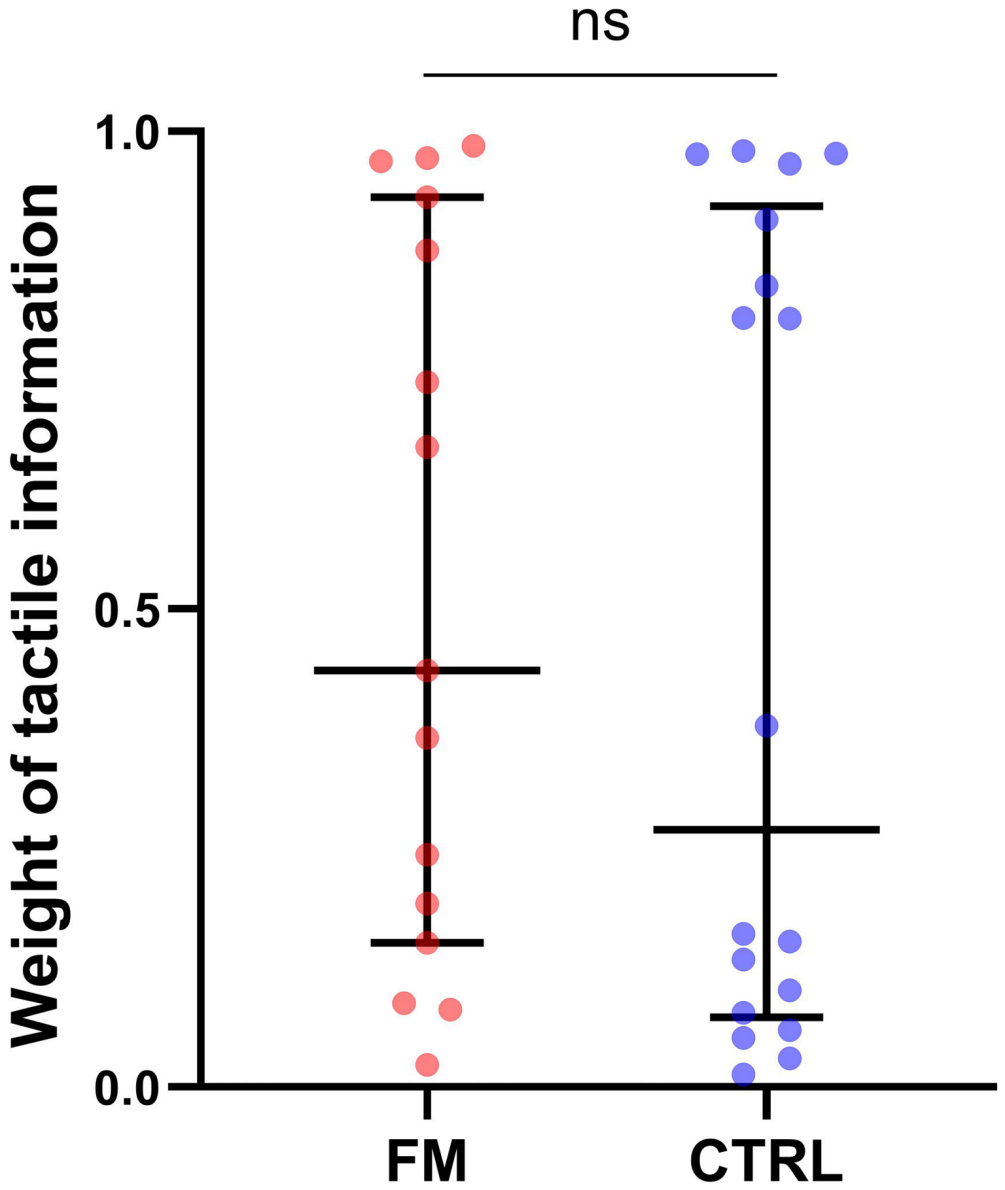
Weight of the tactile information in the visuotactile percept, for each group. Each dot corresponds to a participant of the FM (red) or of the CTRL (blue) group. The middle line represents the median and the errors bars the interquartile range. FM, participants with fibromyalgia; CTRL, pain-free participants; ns, non-significant.

**Figure 6:**
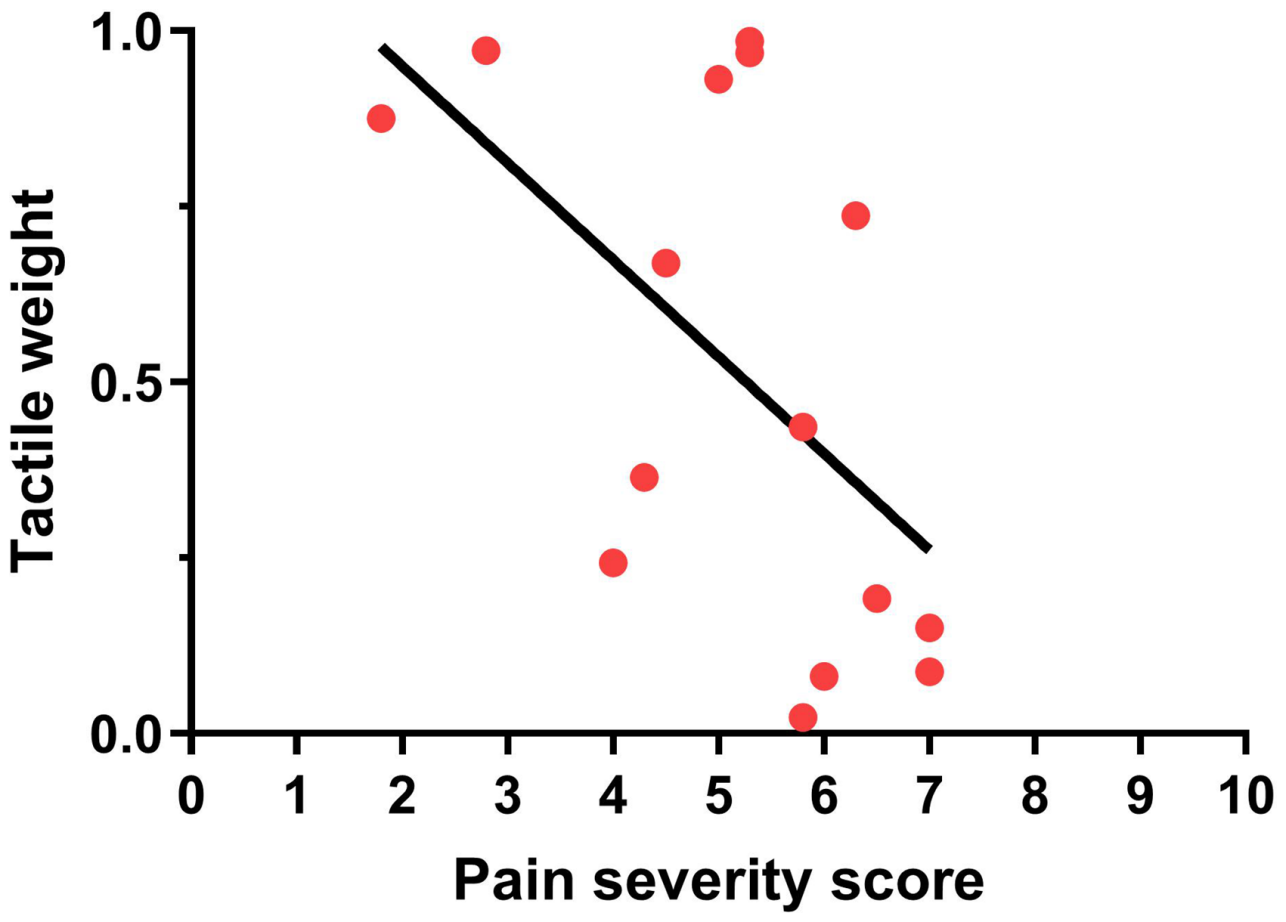
Relationship between the pain severity score of the BPI and the weight attributed to tactile information in participants with FM.

For the weight of the tactile information in the visuotactile percept (**Objective 4**), results of the Mann–Whitney U’s test demonstrated no significant difference between the groups (FM: median = 0.56, IQR = 0.72; CTRL: median = 0.33, IQR = 0.83; *p* = 0.61; see Figure 5). However, this result is tainted by a high between-subject variability. Some participants seemed to have a sensory preference for visual information and some for tactile information, which created a bimodal distribution. We observed this bimodal distribution in both groups. After inspection, no difference between the two clusters (Tactile-dominant, Visual-dominant) was found in age, diagnosis, tactile sensitivity (as measured with the intensity of electrical stimuli chosen for the task), or difference between the tactile sensitivity of the two sites. For the participants with FM, we explored the parallel between the bimodal distribution of the sensory weighting and the slight bimodality of BP (Figure 3A). We found that a bigger proportion of Tactile-dominant participants displayed a positive BP (100% of the subgroup) compared to the Visual-dominant participants (50% of the subgroup). This was not observed for CTRL participants: BP was positive for a similar proportion of participants in both subgroups (90% of the Visual-dominant subgroup and 88% of the Tactile-dominant subgroup). However, for participants with FM, Spearman’s test revealed a significant negative correlation between the pain intensity score of the BPI and the weight attributed to tactile information: the more intense their general pain was, the less they relied on tactile information to perceive the multisensory percept (Spearman’s ρ = −0.551; *p* = 0.033; see Figure 6). Following this observation, we conducted an *a posteriori* analysis of the BP and the BA on the participants with FM with a more intense general pain (as expressed by a BPI severity score > 5; 9 participants out of 15). These participants do not seem to demonstrate more multisensory benefits in precision or in accuracy than participants reporting less intense pain (see Supplementary material).

## 4 Discussion

To our knowledge, this is the first study to assess multisensory integration in individuals with FM and confront it with the MLE model. Results show no intergroup differences in the tactile and visual percepts’ precision and accuracy. Contrary to our hypothesis, participants with FM were as precise and accurate as controls in perceiving the unisensory tactile and visual stimuli. No group differences were observed in the BP and the BA in the multisensory condition. The CTRL group benefitted from access to tactile and visual information, as expressed by increased precision. In contrast, in the FM group, the BP was not different from zero. We observed no benefit in accuracy for either group. According to the MLE model, multisensory integration was optimal for both groups because the observed precision of the multisensory percept was correlated to the precision predicted by the MLE model. Finally, weights attributed to each sensory information were not different between the groups. However, a bimodal distribution of the weights highlighted two clusters of participants based on the preferred sensory information (tactile dominant or visual dominant), regardless of the group. We will first discuss the results of the unisensory conditions. Then, the findings of the multisensory condition will be interpreted in three parts: 1-the multisensory benefits in precision and accuracy; 2-the optimality of the integration according to the MLE; and 3-the weighting of each sensory information in the multisensory percept. Lastly, we will outline some limitations.

In the unisensory conditions, participants with FM were as precise and accurate as CTRL participants in perceiving tactile and visual stimuli despite the tactile alterations reported in this syndrome (Hilgenberg-Sydney et al., 2016; Evdokimov et al., 2019; Martínez et al., 2019). This observation contradicts recent studies showing that participants with FM need longer time intervals between pairs of tactile stimuli to perceive them as separate, compared to pain-free participants (Gunendi et al., 2019; Tertemiz and Tepe, 2022). However, in these studies, the participants with FM reported higher pain intensity than in our sample (mean scores of 7.7/10 and 8.6/10 on two different scales in the study by Tertemiz and Tepe, and of 7.3/10 in the study by Gunendi et al., compared to the BPI pain severity score of 5.4/10 reported in the present study). In the study by Tertemiz and Tepe (2022), tactile detection thresholds were also higher for participants with FM than controls, which was not the case in our study. These differences suggest that our sample of participants with FM was not as severely affected, which could explain the divergent findings. When comparing to other measures of tactile perception in individuals with FM, a lack of intergroup difference in tactile perception is however not uncommon (Lim et al., 2016; Palmer et al., 2019; Menten et al., 2022). These studies included participants with FM with similar intensity of pain as our sample (Menten et al.: 5.45/10; Lim et al.: 57.2/100; Palmer et al.: 5.68/10) and as studies with large samples of participants with FM (Staud et al., 2006: 277 participants with FM with a mean pain of 4.6/10; Steiner et al., 2017: 216 FM with a mean pain intensity of 5.95/10; Serpas et al., 2022: 147 FM, mean pain intensity of 5.96/10). This result is consistent with the influence of pain intensity on unisensory tactile perception.

For both groups, the tactile percept was as precise as the visual percept, confirming that the reliability level was comparable between the two modalities. This is particularly important for multisensory integration studies since an apparent asymmetry of the reliability between the modalities could lead to the exclusive use of the most reliable modality and prevent multisensory integration [a phenomenon called sensory capture (Rohde et al., 2016)].

Results show that the accuracy of the tactile percept was better than the accuracy of the visual percept for both groups. A hypothesis for this result could be the transmission speed difference between tactile and visual information. Tactile information conduction is considered faster than visual information, meaning that a visual stimulation must be triggered slightly before a tactile stimulation for participants to perceive them as simultaneous (Spence et al., 2003; Poliakoff et al., 2006; Keetels and Vroomen, 2011; Godlove et al., 2014). Therefore, tactile information may be more accurate than visual information in a temporal perception task such as the TOJ (Crevecoeur et al., 2016).

In the multisensory condition, the BP was significantly positive for the CTRL group but not for the FM group. Despite this result, we observed no statistically significant difference in the BP between the groups. Together, these data suggest that the benefit of precision in participants with FM may have been masked by intragroup variability.

The analysis of the BA shows that the accuracy was not better in the multisensory condition compared to the unisensory conditions (i.e., no real benefit in accuracy) for both groups. A trend toward a positive BA was nonetheless observed for the FM group; again, intragroup variability might have prevented the BA to reach significance in this group. We expected to detect a benefit in accuracy for the CTRL group to accompany the benefit in precision observed. However, the inspection of the performance in the unisensory conditions shows that the visual and tactile percepts’ accuracy was already excellent for both groups, leaving little room for improvement. It is also important to note that, contrary to the benefit in precision, a benefit in accuracy is not necessary to show multisensory integration. According to Rohde and colleagues, “noise reduction is the most important hallmark of optimal integration” (Rohde et al., 2016). Thus, an improvement in accuracy was considered as a secondary measure, nonessential to determine multisensory integration. A difference of M in the multisensory condition compared to the unisensory conditions can also be used as a complementary measure of sensory weighting: the closer the multisensory M is to one of the unisensory M, the larger the weight of the sensory information is Bultitude and Petrini (2021). In a recent study, participants pointed to visual targets using either visual, somatosensory, or visual and somatosensory information (Bultitude and Petrini, 2021). Healthy participants showed no difference between the M of the multisensory percept compared to the M of the unisensory percepts, which was interpreted as an absence of difference in the weighting of visual and somatosensory information (Bultitude and Petrini, 2021). This also provides evidence for an absence of benefit in accuracy, even though an improvement of precision was observed (Bultitude and Petrini, 2021).

When the precision observed in the multisensory condition was compared to the precision predicted by the MLE model, a positive correlation was found for both groups, which suggests an optimal integration of visual and tactile information. Here, optimality refers to an appropriate integration of the visuotactile stimuli, as defined by the MLE model. Given a certain precision of the unisensory percepts, the model predicts the precision of the multisensory percept. The correlation shows that that observed visuotactile precision follows that predicted by the model.

To summarize, the results in the multisensory condition point to an optimal visuotactile integration for perception in individuals with FM and pain-free controls. This integration was reflected by an improvement in the precision (non-significant for participants with FM) and a positive correlation between the observed visuotactile precision and the precision predicted by the MLE model. This conclusion is supported by studies testing multisensory integration in individuals with chronic pain by generating body illusions. For example, in the rubber hand illusion, participants feel a tactile stroke on their hidden hand while seeing a rubber hand being stroked simultaneously (Botvinick and Cohen, 1998; Tsakiris and Haggard, 2005). The integration of the tactile information felt on the hand with the visual information of the stroked rubber hand leads most pain-free participants to feel that the rubber hand belongs to them (Botvinick and Cohen, 1998; Lloyd, 2007). This illusion type depends on a functioning visuotactile integration (Limanowski, 2021). Several studies found that participants with chronic pain also experience these illusions, suggesting that multisensory integration is not completely disrupted (McCabe et al., 2007; Reinersmann et al., 2013; Don et al., 2016; Brun et al., 2018; Martínez et al., 2018). The intensity of the illusion, however, varied across these studies, leading some authors to suspect a different weighting of the sensory modalities.

Our study showed no such difference since the weighting of visual and tactile information was comparable between the FM and the CTRL groups. Considering the absence of alteration of unisensory integration of tactile information in the FM group, this result is consistent with optimal multisensory integration. Tactile information was not less precise or less accurate in participants with FM and, therefore, was not weighted differently compared to CTRL participants. In fact, for participants with FM, it seemed advantageous to use tactile information more, since a bigger proportion of participants with FM who showed a dominance for tactile information displayed a positive BP. This perceptual benefit of weighting tactile information more was not observed for CTRL participants.

In the group with FM, tactile weight was correlated with general pain intensity, meaning that, in the multisensory condition, participants who experienced more intense pain relied less on tactile information to perceive the visuotactile stimuli. This points to an influence of pain on tactile weight within the FM group. This only partly agrees with our hypothesis since we observed no intergroup difference in sensory weighting. Congruent results were reported in a body-illusion paradigm (Brun et al., 2018). Participants with FM experienced a stronger illusion compared to pain-free controls, and this group difference was explained by the intensity of their pain (Brun et al., 2018). Thus, FM may not be automatically associated with smaller tactile weights, but the presence of moderate to severe pain might be decisive.

Mean sensory weights should nevertheless be interpreted carefully because the distribution of the weights revealed two clusters of participants, regardless of the group: a tactile-dominant cluster and a visual-dominant cluster. Although the participants integrated visual and tactile information (i.e., no sensory capture), a minority relied on both modalities symmetrically or quasi-symmetrically (i.e., weights in the 0.4 to 0.6 range). This weighting strategy was not explained by demographic characteristics or tactile sensitivity differences (as measured by the tactile detection threshold). A similar weight range was reported in a few other multisensory studies involving (Brun et al., 2018) a diverse clinical population (Brun et al., 2018) or pain-free participants (Maiworm and Röder, 2011). In a visuoauditory task, Maiworm and Röder (2011) reported that about half of the participants integrated information optimally, whereas the other half did not. Thus, they divided participants into an Optimal group, attributing a smaller weight to the auditory signal, and a Suboptimal group, relying more on the auditory signal (Maiworm and Röder, 2011). The authors conclude on an individual’s “hard-coded preference for certain modalities under particular circumstances” (Maiworm and Röder, 2011). This top-down predisposition [sometimes referred to as a prior (Limanowski, 2021)] could originate from an attentional bias towards one modality, which would lead to faster processing times and higher weights (Spence et al., 2001; Werkhoven et al., 2009; Maiworm and Röder, 2011; Limanowski, 2021). Another explanation for the bimodal distribution observed in the FM and CTRL groups could lie in a difference in sensory precision between the clusters. We observed no link between the weighting strategy and tactile sensitivity; thus, variations in visual abilities might have played a role. All participants had normal or corrected-to-normal vision and scored at least 60% in the V condition, but finer inter-subject disparities could have influenced sensory weighting (Ernst and Banks, 2002; Helbig and Ernst, 2007; Fetsch et al., 2012).

A technical limitation of the present study was the inability to customize tactile stimuli intensity between the two sites. The chosen stimulation intensity was readily detectable, though not uncomfortable or painful for all participants, finer sensation differences were still present between Site 1 and Site 2 for some participants. This observation did not lead to differences in the preferred site between the groups (in the T condition, the frequency of answers “Site 1” was 0.52 for both groups), which limits its potential bias on the TOJ results. Another limitation is the variability of the group with FM. Though variability seems intrinsic to FM (Wilson et al., 2009; Rehm et al., 2010; Yim et al., 2017; Bartley et al., 2018) our group’s relatively small sample size may have exacerbated this variability. Our sample size was based on pragmatic considerations, given the prevalence of this population and the associated symptoms (severe fatigue, trouble concentrating over long periods, difficulty moving or walking, etc.). Moreover, a study with a sample size similar as ours (15 participants with FM and 15 pain-free controls) showed statistically significant intergroup differences in the temporal discrimination of somatosensory stimuli (Gunendi et al., 2019). Finally, excluding participants whose unisensory performance was too high or too low was necessary to ensure proper multisensory integration [the elimination rate can reach over half of the participants in some multisensory integration studies (Kostaki and Vatakis, 2018)].

In conclusion, we found no difference in multisensory integration between participants with FM and pain-free participants. Both groups integrated visuotactile stimuli optimally, resulting in a more precise visuotactile percept than the visual or tactile percepts (although intragroup variability might have prevented the improvement in precision to reach significance in the group with FM). The mean weighting of the visual and the tactile information in the visuotactile percept was not different between the groups either, which is consistent with the fact that unisensory perception of the visual and tactile percepts was not different between the groups. In the group with FM, the weight attributed to tactile information was correlated to pain intensity, with a smaller weight given by participants with more intense pain. Overall, these findings point to an absence of alterations in multisensory integration in individuals with FM. However, future research should further investigate the role intense pain may play in weighting tactile information.

## Data availability statement

The raw data supporting the conclusions of this article will be made available by the authors, without undue reservation.

## Ethics statement

The studies involving humans were approved by CIUSSS de la Capitale-Nationale, Quebec City, Canada. The studies were conducted in accordance with the local legislation and institutional requirements. The participants provided their written informed consent to participate in this study.

## Author contributions

TA: Conceptualization, Formal analysis, Investigation, Methodology, Visualization, Writing – original draft. MS: Funding acquisition, Supervision, Writing – review & editing. CM: Conceptualization, Funding acquisition, Supervision, Writing – review & editing.

## References

[ref1] Achenbach J. Tran A. T. Jaeger B. Kapitza K. Bernateck M. Karst M. (2020). Quantitative sensory testing in patients with multisomatoform disorder with chronic pain as the leading bodily symptom-a matched case-control study. Pain Med. 21, e54–e61. doi: 10.1093/pm/pnz195, PMID: 31578559

[ref2] Alais D. Burr D. (2004). The ventriloquist effect results from near-optimal bimodal integration. Curr. Biol. 14, 257–262. doi: 10.1016/j.cub.2004.01.029, PMID: 14761661

[ref3] Bartley E. J. Robinson M. E. Staud R. (2018). Pain and fatigue variability patterns distinguish subgroups of fibromyalgia patients [internet]. J. Pain 19, 372–381. doi: 10.1016/j.jpain.2017.11.014 29253551 PMC5869098

[ref4] Botvinick M. Cohen J. (1998). Rubber hands “feel” touch that eyes see. Nature 391:756. doi: 10.1038/35784, PMID: 9486643

[ref5] Branco J. C. Bannwarth B. Failde I. Abello Carbonell J. Blotman F. Spaeth M.. (2010). Prevalence of fibromyalgia: a survey in five European countries. Semin Arthritis Rheum 39, 448–453. doi: 10.1016/j.semarthrit.2008.12.003, PMID: 19250656

[ref6] Bresciani J. P. Dammeier F. Ernst M. O. (2006). Vision and touch are automatically integrated for the perception of sequences of events. J. Vis. 6, 554–564. doi: 10.1167/6.5.2, PMID: 16881788

[ref7] Brun C. Giorgi N. Pinard A. M. Gagné M. McCabe C. S. Mercier C. (2019). Exploring the relationships between altered body perception, limb position sense, and limb movement sense in complex regional pain syndrome. J. Pain 20, 17–27. doi: 10.1016/j.jpain.2018.07.008, PMID: 30099211

[ref8] Brun C. Mercier C. Grieve S. Palmer S. Bailey J. McCabe C. S. (2018). Sensory disturbances induced by sensorimotor conflicts are higher in complex regional pain syndrome and fibromyalgia compared to arthritis and healthy people, and positively relate to pain intensity. Europ. J. Pain 23, 483–494. doi: 10.1002/ejp.1322 30288850

[ref9] Bultitude J. H. Petrini K. (2021). Altered visuomotor integration in complex regional pain syndrome. Behav. Brain Res. 397:112922. doi: 10.1016/j.bbr.2020.112922, PMID: 32971196

[ref10] Chancel M. Blanchard C. Guerraz M. Montagnini A. Kavounoudias A. (2016). Optimal visuotactile integration for velocity discrimination of self-hand movements. J. Neurophysiol. 116, 1522–1535. doi: 10.1152/jn.00883.2015, PMID: 27385802 PMC5040371

[ref11] Chancel M. Ehrsson H. H. (2023). Proprioceptive uncertainty promotes the rubber hand illusion. Cortex 165, 70–85. doi: 10.1016/j.cortex.2023.04.005, PMID: 37269634 PMC10284257

[ref12] Chouvardas V. G. Miliou A. N. Hatalis M. K. (2008). Tactile displays: overview and recent advances. Displays 29, 185–194. doi: 10.1016/j.displa.2007.07.003

[ref13] Clark T. K. Merfeld D. M. (2021). Statistical approaches to identifying lapses in psychometric response data. Psychon. Bull. Rev. 28, 1433–1457. doi: 10.3758/s13423-021-01876-2, PMID: 33825094

[ref14] Cleeland C. S. (1991). Brief Pain Inventory Short Form (BPI-SF) [Database record]. APA PsycTests. doi: 10.1037/t04175-000

[ref15] Crevecoeur F. Munoz D. P. Scott S. H. Crevecoeur F. Munoz D. P. Scott S. H.. (2016). Dynamic multisensory integration: somatosensory speed trumps visual accuracy during feedback control. J. Neurosci. 36, 8598–8611. doi: 10.1523/JNEUROSCI.0184-16.2016, PMID: 27535908 PMC6601898

[ref16] de Vignemont F. Ehrsson H. H. Haggard P. (2005). Bodily illusions modulate tactile perception. Curr. Biol. 15, 1286–1290. doi: 10.1016/j.cub.2005.06.067 16051171

[ref17] Dieguez S. Lopez C. (2017). The bodily self: insights from clinical and experimental research. Ann. Phys. Rehabil. Med. 60, 198–207. doi: 10.1016/j.rehab.2016.04.007 27318928

[ref18] Don S. Voogt L. Meeus M. De Kooning M. Nijs J. (2016). Sensorimotor incongruence in people with musculoskeletal pain: a systematic review. Pain Pract. 17, 115–128. doi: 10.1111/papr.12456, PMID: 27206852

[ref19] Ernst M. O. Banks M. S. (2002). Humans integrate visual and haptic information in a statistically optimal fashion. Nature 415, 429–433. doi: 10.1038/415429a, PMID: 11807554

[ref20] Ernst M. O. Bülthoff H. H. (2004). Merging the senses into a robust percept. Trends Cogn. Sci. 8, 162–169. doi: 10.1016/j.tics.2004.02.002, PMID: 15050512

[ref21] Evdokimov D. Frank J. Klitsch A. Unterecker S. Warrings B. Serra J.. (2019). Reduction of skin innervation is associated with a severe fibromyalgia phenotype. Ann. Neurol. 86, 504–516. doi: 10.1002/ana.25565, PMID: 31376174

[ref22] Fetsch C. R. Deangelis G. C. Angelaki D. E. (2010). Visual-vestibular cue integration for heading perception: applications of optimal cue integration theory. Eur. J. Neurosci. 31, 1721–1729. doi: 10.1111/j.1460-9568.2010.07207.x, PMID: 20584175 PMC3108057

[ref23] Fetsch C. R. Pouget A. Deangelis G. C. Angelaki D. E. (2012). Neural correlates of reliability-based cue weighting during multisensory integration. Nat. Neurosci. 15, 146–154. doi: 10.1038/nn.2983, PMID: 22101645 PMC3398428

[ref24] Geber C. Magerl W. Fondel R. Fechir M. Rolke R. Vogt T.. (2008). Numbness in clinical and experimental pain—a cross-sectional study exploring the mechanisms of reduced tactile function. Pain 139, 73–81. doi: 10.1016/j.pain.2008.03.006, PMID: 18423989

[ref25] Gepshtein S. Banks M. S. (2003). Viewing geometry determines how vision and haptics combine in size perception. Curr. Biol. 13, 483–488. doi: 10.1016/S0960-9822(03)00133-7, PMID: 12646130

[ref26] Godlove J. M. Whaite E. O. Batista A. P. (2014). Comparing temporal aspects of visual, tactile, and microstimulation feedback for motor control. J. Neural Eng. 11:046025. doi: 10.1088/1741-2560/11/4/046025, PMID: 25028989 PMC4156317

[ref27] Gunendi Z. Polat M. Vuralli D. Cengiz B. (2019). Somatosensory temporal discrimination is impaired in fibromyalgia. J. Clin. Neurosci. 60, 44–48. doi: 10.1016/j.jocn.2018.10.067, PMID: 30528354

[ref28] Harvie D. S. Edmond-Hank G. Smith A. D. (2017). Tactile acuity is reduced in people with chronic neck pain. Musculoskelet Sci. Pract. 33, 61–66. doi: 10.1016/j.msksp.2017.11.009, PMID: 29180111

[ref29] Häuser W. Ablin J. Fitzcharles M. A. Littlejohn G. Luciano J. V. Usui C.. (2015). Fibromyalgia. Nat. Rev. Dis. Primers 1, 1–16. doi: 10.1038/nrdp.2015.22 27189527

[ref30] Häuser W. Fitzcharles M. A. (2018). Facts and myths pertaining to fibromyalgia. Dialogues Clin. Neurosci. 20, 53–62. doi: 10.31887/DCNS.2018.20.1/whauser, PMID: 29946212 PMC6016048

[ref31] Helbig H. B. Ernst M. O. (2007). Optimal integration of shape information from vision and touch. Exp. Brain Res. 179, 595–606. doi: 10.1007/s00221-006-0814-y, PMID: 17225091

[ref32] Helbig H. B. Ernst M. O. Ricciardi E. Pietrini P. Thielscher A. Mayer K. M.. (2012). The neural mechanisms of reliability weighted integration of shape information from vision and touch. NeuroImage 60, 1063–1072. doi: 10.1016/j.neuroimage.2011.09.072, PMID: 22001262

[ref33] Hilgenberg-Sydney P. B. Kowacs P. A. Conti P. C. R. (2016). Somatosensory evaluation in dysfunctional syndrome patients. J. Oral Rehabil. 43, 89–95. doi: 10.1111/joor.12344 26337788

[ref34] Hochman J. R. Davis A. M. Elkayam J. Gagliese L. Hawker G. A. (2013). Neuropathic pain symptoms on the modified painDETECT correlate with signs of central sensitization in knee osteoarthritis. Osteoarthr. Cartil. 21, 1236–1242. doi: 10.1016/j.joca.2013.06.023, PMID: 23973136

[ref35] Keetels M. Vroomen J. Perception of synchrony between the senses. In (2011). p. 147–178. Available at: http://www.crcnetbase.com/doi/abs/10.1201/9781439812174-12 22593865

[ref36] Kim H. Mawla I. Lee J. Gerber J. Walker K. Kim J.. (2020). Reduced tactile acuity in chronic low back pain is linked with structural neuroplasticity in primary somatosensory cortex and is modulated by acupuncture therapy. NeuroImage 217:116899. doi: 10.1016/j.neuroimage.2020.116899, PMID: 32380138 PMC7395964

[ref37] Kostaki M. Vatakis A. (2018). “Temporal order and synchrony judgments: a primer for students” in Timing and time perception: Procedures, measures, and applications. Brill. 233–262.

[ref38] Lackner J. R. (1988). Some proprioceptive influences on the perceptual representation of body shape and orientation. Brain 111, 281–297. doi: 10.1093/brain/111.2.281, PMID: 3378137

[ref39] Lewis J. S. Kersten P. McCabe C. S. McPherson K. M. Blake D. R. (2007). Body perception disturbance: a contribution to pain in complex regional pain syndrome (CRPS). Pain 133, 111–119. doi: 10.1016/j.pain.2007.03.013, PMID: 17509761

[ref40] Ley I. Haggard P. Yarrow K. (2009). Optimal integration of auditory and Vibrotactile information for judgments of temporal order. J. Exp. Psychol. Hum. Percept. Perform. 35, 1005–1019. doi: 10.1037/a0015021, PMID: 19653745

[ref41] Lim M. Roosink M. Kim J. S. Kim H. W. H. A. H. W. H. A. H. W. H. A. Lee E. B. Son K. M.. (2016). Augmented pain processing in primary and secondary somatosensory cortex in fibromyalgia: a magnetoencephalography study using intra-epidermal electrical stimulation. PLoS One 11:e0151776. doi: 10.1371/journal.pone.0151776 26992095 PMC4798786

[ref42] Limanowski J. (2021). Precision control for a flexible body representation. Neurosci. Biobehav. Rev. 134, 1–18. doi: 10.1016/j.neubiorev.2021.10.023 34736884

[ref43] Lloyd D. M. (2007). Spatial limits on referred touch to an alien limb may reflect boundaries of visuo-tactile peripersonal space surrounding the hand. Brain Cogn. 64, 104–109. doi: 10.1016/j.bandc.2006.09.013, PMID: 17118503

[ref44] López-de-Uralde-Villanueva I. Tostado-Haro I. Noval-Granda B. Ferrer-Peña R. Del Corral T. (2020). Widespread impairment of tactile spatial acuity and sensory-motor control in patients with chronic nonspecific neck pain with neuropathic features. Musculoskelet. Sci. Pract. 47:102138. doi: 10.1016/j.msksp.2020.102138 32148331

[ref45] Lotze M. Moseley G. L. (2007). Role of distorted body image in pain. Curr. Rheumatol. Rep. 9, 488–496. doi: 10.1007/s11926-007-0079-x, PMID: 18177603

[ref46] Maier C. Baron R. Tölle T. R. Binder A. Birbaumer N. Birklein F.. (2010). Quantitative sensory testing in the German research network on neuropathic pain (DFNS): somatosensory abnormalities in 1236 patients with different neuropathic pain syndromes. Pain 150, 439–450. doi: 10.1016/j.pain.2010.05.002, PMID: 20627413

[ref47] Maiworm M. Röder B. (2011). Suboptimal auditory dominance in audiovisual integration of temporal cues. Tsinghua Sci. Technol. 16, 121–132. doi: 10.1016/S1007-0214(11)70019-0

[ref48] Marcus P. L. Fuglevand A. J. (2009). Perception of electrical and mechanical stimulation of the skin: implications for electrotactile feedback. J. Neural Eng. 6:066008. doi: 10.1088/1741-2560/6/6/066008, PMID: 19918109 PMC7738194

[ref49] Martínez E. Aira Z. Buesa I. Aizpurua I. Rada D. Azkue J. J. (2018). Embodied pain in fibromyalgia: disturbed somatorepresentations and increased plasticity of the body schema. PLoS One 13, 1–17. doi: 10.1371/journal.pone.0194534 PMC5889164 29624596

[ref50] Martínez E. Guillen V. Buesa I. Azkue J. J. (2019). A distorted body Schema and susceptibility to experiencing anomalous somatosensory sensations in fibromyalgia syndrome. Clin. J. Pain 35, 887–893. doi: 10.1097/AJP.0000000000000754, PMID: 31433318

[ref51] McCabe C. S. Cohen H. Blake D. R. (2007). Somaesthetic disturbances in fibromyalgia are exaggerated by sensory—motor conflict: implications for chronicity of the disease? Rheumatology 46, 1587–1592. doi: 10.1093/rheumatology/kem204, PMID: 17767000

[ref52] Menten L. A. Franco K. F. M. Franco Y. R. S. Miyamoto G. C. Reis F. J. J. Cabral C. M. N. (2022). Do patients with fibromyalgia have body image and tactile acuity distortion? Pain Pract. 22, 678–687. doi: 10.1111/papr.13153, PMID: 36345889

[ref53] Morein-Zamir S. Soto-Faraco S. Kingstone A. (2003). Auditory capture of vision: examining temporal ventriloquism. Cogn. Brain Res. 17, 154–163. doi: 10.1016/S0926-6410(03)00089-2, PMID: 12763201

[ref54] Moseley G. L. (2008). I can’t find it! Distorted body image and tactile dysfunction in patients with chronic back pain. Pain 140, 239–243. doi: 10.1016/j.pain.2008.08.001, PMID: 18786763

[ref55] Nardini M. Jones P. Bedford R. Braddick O. (2008). Development of Cue integration in human navigation. Curr. Biol. 18, 689–693. doi: 10.1016/j.cub.2008.04.021, PMID: 18450447

[ref56] Noguchi K. Gel Y. R. Brunner E. Konietschke F. (2012). nparLD: an R software package for the nonparametric analysis of longitudinal data in factorial experiments. J. Stat. Softw. 50, 1–23. doi: 10.18637/jss.v050.i1225317082

[ref57] Oldfield R. C. (1971). The assessment and analysis of handedness: the Edinburgh inventory. Neuropsychologia 9, 97–113. doi: 10.1016/0028-3932(71)90067-4 5146491

[ref58] Palmer S. Bailey J. Brown C. Jones A. McCabe C. S. (2019). Sensory function and pain experience in arthritis, complex regional pain syndrome, fibromyalgia syndrome and healthy volunteers: a cross-sectional study. Clin. J. Pain 35, 894–900. doi: 10.1097/AJP.0000000000000751, PMID: 31408010

[ref59] Peltz E. Seifert F. Lanz S. Müller R. Maihöfner C. (2011). Impaired hand size estimation in CRPS. J. Pain 12, 1095–1101. doi: 10.1016/j.jpain.2011.05.001, PMID: 21741321

[ref60] Pleger B. Ragert P. Schwenkreis P. Förster A. F. Wilimzig C. Dinse H.. (2006). Patterns of cortical reorganization parallel impaired tactile discrimination and pain intensity in complex regional pain syndrome. NeuroImage 32, 503–510. doi: 10.1016/j.neuroimage.2006.03.045, PMID: 16753306

[ref61] Poliakoff E. Shore D. I. Lowe C. Spence C. (2006). Visuotactile temporal order judgments in ageing. Neurosci. Lett. 396, 207–211. doi: 10.1016/j.neulet.2005.11.034, PMID: 16356634

[ref62] Ransil B. J. Schachter S. C. (1994). Test-retest reliability of the Edinburgh handedness inventory and global handedness preference measurements, and their correlation. Percept. Mot. Skills 79, 1355–1372. doi: 10.2466/pms.1994.79.3.1355, PMID: 7899020

[ref63] Regenbogen C. Seubert J. Johansson E. Finkelmeyer A. Andersson P. Lundström J. N. (2018). The intraparietal sulcus governs multisensory integration of audiovisual information based on task difficulty. Hum. Brain Mapp. 39, 1313–1326. doi: 10.1002/hbm.23918, PMID: 29235185 PMC6866436

[ref64] Rehm S. E. Koroschetz J. Gockel U. Brosz M. Freynhagen R. Tölle T. R.. (2010). A cross-sectional survey of 3035 patients with fibromyalgia: subgroups of patients with typical comorbidities and sensory symptom profiles. Rheumatology 49, 1146–1152. doi: 10.1093/rheumatology/keq066, PMID: 20236955

[ref65] Reinersmann A. Landwehrt J. Krumova E. K. Peterburs J. Ocklenburg S. Güntürkün O.. (2013). The rubber hand illusion in complex regional pain syndrome: preserved ability to integrate a rubber hand indicates intact multisensory integration. Pain 154, 1519–1527. doi: 10.1016/j.pain.2013.03.039, PMID: 23706626

[ref66] Rohde M. Van Dam L. C. J. Ernst M. O. (2016). Statistically optimal multisensory cue integration: a practical tutorial. Multisens. Res. 29, 279–317, PMID: 29384605 10.1163/22134808-00002510

[ref67] Rommel O. Malin J. P. Zenz M. Ènig W. J. (2001). Quantitative sensory testing, neurophysiological and psychological examination in patients with complex regional pain syndrome and hemisensory deficits. Pain Suppl. 93, 279–293. doi: 10.1016/S0304-3959(01)00332-3, PMID: 11514087

[ref68] Ronsse R. Miall R. C. Swinnen S. P. (2009). Multisensory integration in dynamical behaviors: maximum likelihood estimation across bimanual skill learning. J. Neurosci. 29, 8419–8428. doi: 10.1523/JNEUROSCI.5734-08.2009, PMID: 19571132 PMC5116379

[ref001] RStudio Team (2023). RStudio: Integrated Development for R. RStudio, PBC. Boston, MA. Available at: http://www.rstudio.com/.

[ref69] Scandola M. Pietroni G. Landuzzi G. Polati E. Schweiger V. Moro V. (2022). Bodily illusions and motor imagery in fibromyalgia. Front. Hum. Neurosci. 15:798912. doi: 10.3389/fnhum.2021.798912, PMID: 35126075 PMC8811121

[ref70] Serpas D. G. Zettel-Watson L. Cherry B. J. (2022). Pain intensity and physical performance among individuals with fibromyalgia in mid-to-late life: the influence of depressive symptoms. J. Health Psychol. 27, 1723–1737. doi: 10.1177/13591053211009286, PMID: 33840234

[ref71] Sexton B. M. Liu Y. Block H. J. (2019). Increase in weighting of vision vs. proprioception associated with force field adaptation. Sci. Rep. 9:10167. doi: 10.1038/s41598-019-46625-7, PMID: 31308399 PMC6629615

[ref72] Sobeeh M. G. Hassan K. A. da Silva A. G. Youssef E. F. Fayaz N. A. Mohammed M. M. (2023). Pain mechanisms in complex regional pain syndrome: a systematic review and meta-analysis of quantitative sensory testing outcomes. J. Orthop. Surg. Res. 18:2. doi: 10.1186/s13018-022-03461-2, PMID: 36593515 PMC9806919

[ref73] Spence C. Baddeley R. Zampini M. James R. Shore D. I. (2003). Multisensory temporal order judgments: when two locations are better than one. Percept. Psychophys. 65, 318–328. doi: 10.3758/BF03194803, PMID: 12713247

[ref74] Spence C. Shore D. I. Klein R. M. (2001). Multisensory prior entry. J. Exp. Psychol. Gen. 130, 799–832. doi: 10.1037/0096-3445.130.4.799, PMID: 11757881

[ref75] Staud R. Vierck C. J. Robinson M. E. Price D. D. (2006). Overall fibromyalgia pain is predicted by ratings of local pain and pain-related negative affect—possible role of peripheral tissues. Rheumatology 45, 1409–1415. doi: 10.1093/rheumatology/kel121, PMID: 16621922

[ref76] Steiner J. L. Bigatti S. M. Slaven J. E. Ang D. C. (2017). The complex relationship between pain intensity and physical functioning in fibromyalgia: the mediating role of depression. J. Appl. Biobehav. Res. 22:12079. doi: 10.1111/jabr.12079, PMID: 29527113 PMC5839337

[ref77] Tertemiz O. F. Tepe N. (2022). Is two-point discrimination test a new diagnostic method for the diagnosis of fibromyalgia? Noropsikiyatri Arsivi. 59, 87–90. doi: 10.29399/npa.27245, PMID: 35685043 PMC9142026

[ref78] Tsakiris M. Haggard P. (2005). The rubber hand illusion revisited: Visuotactile integration and self-attribution. J. Exp. Psychol. Hum. Percept. Perform. 31, 80–91. doi: 10.1037/0096-1523.31.1.80, PMID: 15709864

[ref79] Üçeyler N. Zeller J. Kewenig S. Kittel-Schneider S. Fallgatter A. J. Sommer C. (2015). Increased cortical activation upon painful stimulation in fibromyalgia syndrome. BMC Neurol. 15, 1–11. doi: 10.1186/s12883-015-0472-4 26486985 PMC4618366

[ref80] Valenzuela-Moguillansky C. (2013). An exploration of the bodily experience of persons suffering from fibromyalgia. Constr. Found. 8, 339–350.

[ref81] Veale J. F. (2014). Edinburgh handedness inventory—short form: a revised version based on confirmatory factor analysis. Laterality 19, 164–177. doi: 10.1080/1357650X.2013.783045 23659650

[ref82] Werkhoven P. J. Van Erp J. B. F. Philippi T. G. (2009). Counting visual and tactile events: the effect of attention on multisensory integration. Atten. Percept. Psychophys. 71, 1854–1861. doi: 10.3758/APP.71.8.1854, PMID: 19933568

[ref83] Westermann A. Rönnau A. K. Krumova E. Regeniter S. Schwenkreis P. Rolke R.. (2011). Pain-associated mild sensory deficits without hyperalgesia in chronic non-neuropathic pain. Clin. J. Pain 27, 782–789. doi: 10.1097/AJP.0b013e31821d8fce, PMID: 21642846

[ref84] Wilson H. D. Starz T. W. Robinson J. P. Turk D. C. (2009). Heterogeneity within the fibromyalgia population: theoretical implications of variable tender point severity ratings. J. Rheumatol. 36, 2795–2801. doi: 10.3899/jrheum.090432, PMID: 19918043

[ref85] Wolfe F. Brähler E. Hinz A. Häuser W. (2013). Fibromyalgia prevalence, somatic symptom reporting, and the dimensionality of polysymptomatic distress: results from a survey of the general population. Arthritis. Care Res. (Hoboken) 65, 777–785. doi: 10.1002/acr.21931, PMID: 23424058

[ref86] Wolfe F. Clauw D. J. Fitzcharles M. A. Goldenberg D. L. Häuser W. Katz R. L.. (2016). 2016 revisions to the 2010/2011 fibromyalgia diagnostic criteria. Semin Arthritis Rheum 46, 319–329. doi: 10.1016/j.semarthrit.2016.08.012, PMID: 27916278

[ref87] Wolfe F. Clauw D. J. Fitzcharles M. A. Goldenberg D. L. Katz R. S. Mease P.. (2010). The American College of Rheumatology preliminary diagnostic criteria for fibromyalgia and measurement of symptom severity. Arthritis Care Res. (Hoboken) 62, 600–610. doi: 10.1002/acr.20140, PMID: 20461783

[ref88] Wolfe F. Smythe H. A. Yunus M. B. Bennett R. M. Bombardier C. Goldenberg D. L.. (1990). The American College of Rheumatology 1990 criteria for the classification of fibromyalgia. Report of the multicenter criteria committee. Arth. Rheum 33, 160–172. doi: 10.1002/art.1780330203 2306288

[ref89] Yem V. Kajimoto H. (2017). Comparative evaluation of tactile sensation by electrical and mechanical stimulation. IEEE Trans. Haptics. 10, 130–134. doi: 10.1109/TOH.2016.2605084, PMID: 28113382

[ref90] Yim Y. R. Lee K. E. Park D. J. Kim S. H. Nah S. S. Lee J. H.. (2017). Identifying fibromyalgia subgroups using cluster analysis: relationships with clinical variables. Europ. J. Pain 21, 374–384. doi: 10.1002/ejp.935, PMID: 27633925

