# Supplementary material for: Visuotactile integration in individuals with fibromyalgia
Source: Front Hum Neurosci. 2024 May 17;18:1390609. doi: 10.3389/fnhum.2024.1390609 (PMC11140151; doi:10.3389/fnhum.2024.1390609)
Supplement: Supplementary file 1 [file Data_Sheet_1.PDF]

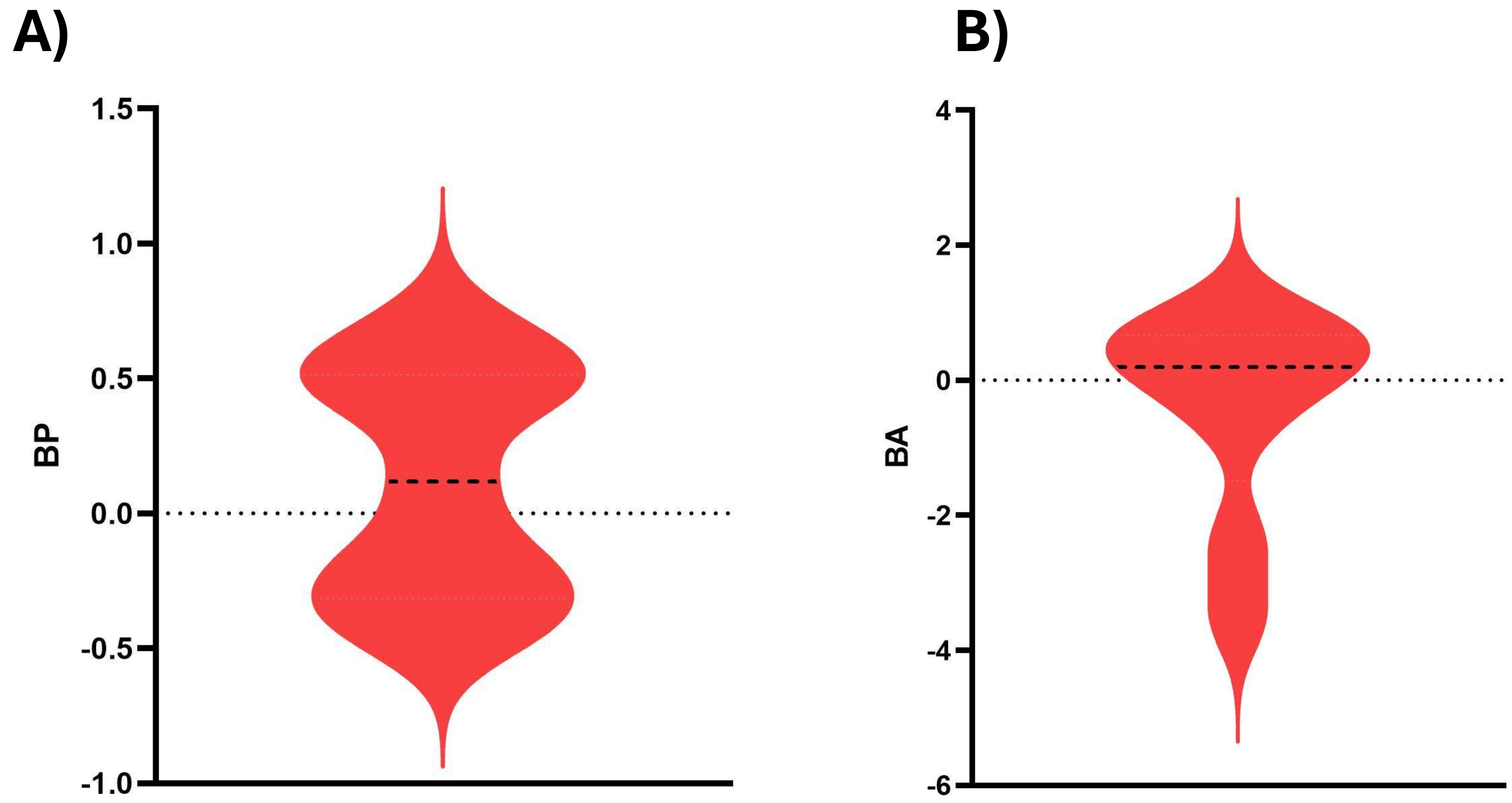

Supplementary material.

Figure 1. Benefits in precision (A) and accuracy (B) for the participants with FM with a BPI Severity score > 5.
